# Perinatal outcomes after increased fetal movement as counted by a fetal movement acceleration measurement recorder

**DOI:** 10.1016/j.eurox.2025.100428

**Published:** 2025-09-11

**Authors:** Eiji Ryo, Hideo Kamata, Keita Yatsuki, Takashi Kosaka, Kazunori Nagasaka

**Affiliations:** Department of Obstetrics and Gynecology, Teikyo University, School of Medicine, 2-11-1, Kaga, Itabashi, Tokyo 173-8606, Japan

**Keywords:** Fetal movement acceleration measurement recorder, FMAM recorder, Fetal non-cephalic presentation, Gross fetal movement, Intrauterine fetal death, Preterm delivery, Perinatal abnormality

## Abstract

**Introduction:**

There is no consensus in perinatal medicine about what increased fetal movement means.

**Objective:**

Our purpose was to study the relationship between perinatal outcomes and an increase in gross fetal movement as counted by an objective method.

**Methods:**

This was a prospective cohort study. A total of 471 pregnant women recorded fetal movement with a fetal movement acceleration measurement recorder at night weekly after 28 weeks. The ratio of 10-second epochs with fetal movement to all epochs was calculated as a fetal movement parameter when movement could be recorded for more than 4 h on one night. If the parameter was above the 90th percentile on the previously made reference curve, it was defined as increased movement. Women who showed the increased fetal movement at least once were classified into a study group, and the other women were classified into a control group. Perinatal abnormalities between the two groups were compared with the Chi-square test or Fisher’s exact test.

**Results:**

A total of 13,931 h were recorded from 390 women, including 74 and 316 women, respectively, in the study and control groups. There were fewer preterm births (1.4 %), non-cephalic presentations at delivery (0 %), and Cesarean deliveries (25.7 %) in the study group than in the control group (15.5 %, 6.0 %, and 39.9 %; p = 0.0003, 0.031, and 0.024, respectively). There were no significant differences in the other perinatal outcomes.

**Conclusion:**

An increase in long-term gross fetal movement has a protective effect against preterm birth, non-cephalic presentation at birth, and Cesarean delivery.

## Highlights

• There is no consensus about what an increase in fetal movement means.
• An increase in fetal movement indicated a protective effect against preterm birth.
• It indicated a protective effect against non-cephalic presentation at birth.
• It indicated a protective effect against Cesarean delivery.

## Introduction

Fetal movement is an important sign of fetal well-being. There is a consensus that a decrease in fetal movement reflects deteriorated fetal conditions, including stillbirth. A widely used biophysical profile scoring system using ultrasonography includes gross fetal and respiratory movement observation, and the lack of movements during a 30-minute time period indicates poor fetal condition [1]. We previously reported that decreased fetal movement counted by an objective method was related to more preterm deliveries, smaller newborns, and hypertensive disorders of pregnancy [2].

On the other hand, there has been no consensus about what an increase in fetal movement means. In 1977, Sadowsky et al [3]. initially reported that a sudden burst of fetal movement was a potential sign of acute fetal distress. Since then, several studies have explored this relationship. Linde et al [4]. reported that 10 % of women suffering stillbirth described extremely vigorous fetal activity followed by no movement. Warland et al [5]. also reported data from a web-based survey of 1714 mothers who had suffered a late stillbirth that 8.5 % reported significantly more fetal movement. Similarly, Whitehead et al [6]. reported that 10–30 % of women who subsequently suffered stillbirth described an episode of excessive fetal movement prior to fetal demise.

On the contrary, Cohen et al [7]. reported in a retrospective cohort study that maternal perception of increased fetal movement was not associated with adverse neonatal outcomes. Sharp et al [8]. reported in a prospective study that there was no evidence of an association between increased fetal movement and adverse neonatal outcomes. In addition, Avraham et al [9]. reported in a prospective cohort and retrospective comparative analysis that subjective sensation of increased fetal movement was not associated with adverse pregnancy outcomes.

In all these studies [3], [4], [5], [6], [7], [8], [9], increased fetal movement was assessed by maternal perception, which is practical and easy to perform; however, it is subjective and includes only short-term observations, which often lead to inaccurate results. This inaccuracy is one of the important reasons why there has been no consensus about the clinical meaning of increased fetal movement.

There used to be no accurate and practical way of counting fetal movements for hours at a time, especially at home; however, we have developed a fetal movement acceleration measurement recorder (FMAM recorder) (ACO CO.,LTD., Tokyo, Japan. https://www.aco-japan.co.jp/company.htlm) that can record gross fetal movement for hours at home [2], [10], [11], [12]. In a basic study [10], gross fetal movements were counted by ultrasonography and maternal abdominal oscillations were simultaneously counted with the FMAM recorder. That study confirmed that the two counts agreed almost perfectly. The FMAM recorder has enabled the long-term monitoring of gross fetal movement.

The purpose of this study was to see what increased fetal movement meant for perinatal outcomes.

## Material and methods

### Subjects

This was a prospective cohort study. A total of 471 singleton pregnant women participated in the study, recorded fetal movement with the FMAM recorder, and delivered a baby between August 2009 and March 2022 at Teikyo University Hospital. Women with major fetal malformation were excluded from the study.

All the mothers were asked to record fetal movement by themselves with the FMAM recorder weekly from 28 to 36 weeks because the accuracy of the FMAM recorder is unreliable before 28 weeks [10]. The outpatient women visited our hospital for routine examinations every two weeks from 28 weeks to 35 weeks and every week from 36 weeks until birth.

### FMAM recorder

The FMAM recorder was explained in detail in our previous studies [2], [10], [11], [12]. The weight is 290 g, and it can be used at home. It has two acceleration sensors: one is a fetal movement sensor that attaches to the mother’s abdominal wall; the other is a mother’s movement sensor attaches to her thigh. The sensitivities of the fetal and maternal sensors are 700 mV/0.1 G and 120 mV/0.1 G, respectively. The fetal movement sensor picks up oscillations of the mother’s abdomen induced by gross fetal movement. However, maternal movement also causes abdominal oscillations; therefore, the recorder is unsuitable when the mother is active. That is why it is used mainly during night sleep. Even during sleep, though, mothers move occasionally. In principle, when the mother’s movement sensor detects no leg movement, and the fetal movement sensor detects oscillations of her abdominal wall, gross fetal movement is judged to have occurred.

### Analysis of fetal movement

The data was transferred from the recorder to a personal computer. Considering the cycle of fetal behavior and maternal sleeping time, we accepted a record as valid only when data could be recorded for more than 4 h in one night. The record was analyzed and fetal movements were counted using a software system, version 1.05 (NoruPro Light Systems Inc., Tokyo, Japan), which was developed especially for the FMAM recorder [12]. A brief description of the system is as follows: 1. The low acceleration signals were filtered and changed to absolute integral values per 50 ms; 2. When the integral values were greater than twice the average amplitude during the 3 s just before and after measurement, they were judged to be positive for acceleration; 3. Any period in which the mother’s sensor detected positive accelerations more than four times per minute was deleted from the data because this usually indicated that the mother was active or awake; 4. Characteristic regular accelerations at 15–20 beats/min detected by the fetal sensor were a sign of fetal hiccups and not counted as fetal movement. 5. When the accelerations of the mother were negative and those of the fetus were positive, fetal movement was judged to have occurred.

The record was divided into intervals (epochs) of 10 s, and an epoch that had a fetal movement was regarded as a positive epoch. The ratio of positive epochs to all epochs during one night was calculated as a parameter of fetal movement.

We previously made fetal movement parameter reference curves depending on gestational week based on observations of women with normal pregnancies and deliveries [11]. When the fetal movement parameter obtained in this study was more than the 90th percentile of the reference curve, it was defined as an increased movement parameter.

### Abnormal perinatal outcomes

Potential abnormal perinatal outcomes included maternal or infant mortality, preterm delivery, non-cephalic presentation at delivery, Cesarean delivery, hypertensive disorder of pregnancy, more than 1000 ml bleeding at delivery, a small- or large- for gestational-age newborn, an Apgar score below 7 points at 1 or 5 min, and umbilical artery pH less than 7.15.

The fetal movement recorded was analyzed after delivery; therefore, the analysis did not affect the management of the pregnancy or delivery.

### Examinations

Women who showed an increased fetal movement parameter at least once were classified into a study group, and the other women were classified into a control group.

First, gestational weeks were classified into 28–30, 31–33, and 34–36 weeks because the fetal movement parameters decrease depending on gestational weeks [11]. Then, we compared the mean of the movement parameters for the study and control groups using Student’s t- test.

Next, as our main investigation, we compared the frequency of abnormal perinatal outcomes for the study and control groups using the Chi-square test or Fisher’s exact test. Significant difference was considered when the p-value was less than 0.05.

## Results

Not all women recorded weekly, and not all data were successfully recorded for more than four hours per night. As a result, we finished with a total of 13,931 h of recording from 390 women. The study group consisted of 74 women, giving us 3.364 h of recording from 521 nights. The control group consisted of 316 women with 10,567 h of recording from 1624 nights.

Table 1 shows the backgrounds of the two groups. The study group consisted of more multipara and more women with lower BMI. Delivery weeks were a bit more in the study group.

**Table 1:** Characteristics.

			Study group (n = 74)		Control group (n = 316)		P value	OR	CI	
Mother										
	Age		34.6 ± 4.0		34.2 ± 5.3		0.561	1.020	0.972	1.085
	Para/non Para		40/34		112/204		0.003	0.467	0.280	0.778
	BMI		20.6 ± 3.0		22.0 ± 3.8		0.004	0.872	0.765	0.996
	<25		68	(91.9)	260	(82.3)	0.042	2.441	1.009	5.904
	25－29		5	(6.8)	40	(12.7)	0.223	0.500	0.190	1.314
	≧ 30		1	(1.4)	16	(5.1)	0.215	0.257	0.034	1.968
	Prior cesarean delivery		8	(10.8)	31	(9.8)	0.830	1.114	0.290	2.535
	Gestational diabetes		3	(4.1)	26	(8.2)	0.324	0.471	0.140	1.601
	Delivery weeks		39.2 ± 1.0		38.5 ± 1.9		0.002	1.437	1.123	1.840
Newborn										
	Male/Female		33/41		156/160		0.460	0.826	0.496	1.373
	Weight(g）		2982.6 ± 410.1		2852.8 ± 544.2		0.135	1.000	0.999	1.001

Data are presented as number(%) or mean±S.D.

The two groups were compared using Student's *t*-test, χ^2^ test, or Fisher's exact test.

BMI = Body mass index, OR = Odds ratio, CI = Confidence interval

The average number of records per woman was 7.04 in the study group and 5.14 in the control group (p = 0.0004). The average length of recording time per record was 6.46 h in the study group and 6.51 h in the control group (p = 0.912).

**Fig. 1:**
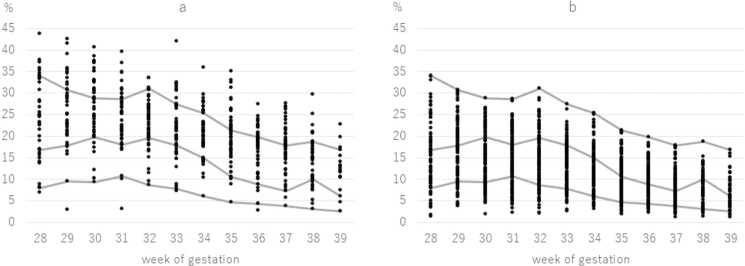
Data plots over the reference curves (Reference 11) from the study group (a) and the control group (b).

Table 2 shows the averages of the fetal movement parameters in both groups. The study group had higher averages than the control group for every gestational period. We assumed that women who showed an increased movement parameter at least once had increased fetal movement overall. Fig. 1 shows data plots over the reference curves (reference 11) from the study group and the control group.

**Table 2:** The mean of the fetal movement parameters.

gestational week	Study group		Control group		P value	OR	CI	
28–30	53	26.2 ± 6.8	201	15.3 ± 6.3	0.001	1.192	1.077	1.320
31–33	66	23.3 ± 5.7	245	13.6 ± 5.5	0.001	1.096	0.965	1.244
34–36	66	19.8 ± 4.2	241	10.7 ± 4.3	0.001	1.368	1.175	1.594

Data are presented as data number and mean±S.D.

The two groups were compared with the Student's *t*-test.

OR = Odds ratio, CI = Confidence interval

The abnormal outcomes are shown in Table 3. Some abnormal outcomes were more frequent than the national average in Japan, probably because our facility is a perinatal medical center treating high-risk pregnancies, and because inpatient women tended to participate in the studies more often than our usual outpatient women. There were no maternal deaths in either group, but there was one stillbirth in the study group. There were fewer preterm births (1.4 %) in the study group than in the control group (15.5 %) (p < 0.001). There were fewer fetal non-cephalic presentations at birth in the study group (0 %) than in the control group (6 %) (p = 0.031). There were fewer Cesarean deliveries (25.7 %) in the study group than in the control group (39.9 %) (p = 0.024). There were no significant differences for other abnormal perinatal outcomes between the two groups.

**Table 3:** The abnormal outcomes.

			Study group (n = 74)		Control group (n = 316)		P value	OR	CI	
Maternal mortality			0	(0)	0	(0)	-	-	-	-
Infant mortality			1	(1.4)	0	(0)	0.190	-	-	-
Preterm delivery			1	(1.4)	49	(15.5)	< 0.001	0.07	0.01	0.55
	< 32week		0	(0.0)	4	(1.3)	1.000	0.00	-	-
	< 34week		0	(0.0)	9	(2.8)	0.218	0.00	-	-
	spontaneous		1	(1.4)	20	(6.3)	0.141	0.20	0.03	1.54
	iatrogenic		0	(0.0)	29	(9.2)	0.003	0.00	-	-
(indications for iatrogenic preterm birth)										
	NRFS		0	(0.0)	7	(2.2)	-	-	-	-
	growth arrest		0	(0.0)	2	(0.6)	-	-	-	-
	Hypertensive disorders of pregnancy		0	(0.0)	13	(4.1)	-	-	-	-
	others		0	(0.0)	7	(2.2)	-	-	-	-
Non-cephalic presentation at delivery			0	(0.0)	19	(6.0)	0.031	0.00	-	-
Cesarean delivery			19	(25.7)	126	(39.9)	0.023	0.52	0.30	0.92
	emergent		9	(12.2)	69	(21.8)	0.061	0.50	0.23	1.05
	planned		10	(13.5)	57	(18.0)	0.353	0.71	0.34	1.47
Hypertensive disorders of pregnancy			3	(4.1)	29	(9.2)	0.081	0.35	0.10	1.17
More than 1000 ml bleeding			12	(16.2)	67	(21.2)	0.337	0.72	0.37	1.41
Light-for-gestational-age newborns			8	(10.8)	43	(13.6)	0.521	0.770	0.35	1.71
	birthweight 3–10th percentile		5	(6.8)	24	(7.6)	0.805	0.88	0.32	2.39
	< 3th percentile		3	(4.1)	19	(6.0)	0.779	0.66	0.19	2.29
Heavy-for-gestational-age newborns			12	(16.2)	39	(12.3)	0.374	1.37	0.68	2.78
Apgar score less than 7										
	1 min		1	(1.4)	15	(4.7)	0.326	0.27	0.04	2.11
	5 min		0	(0.0)	8	(2.5)	0.352	0.00	-	-
Umbilical artery pH less than 7.15			2	(2.7)	2	(0.6)	1.000	0.86	0.18	3.99

Data are presented as number (%).

The two groups were compared using theχ2 test or Fisher's exact test.

NRFS = non-reassuring fetal status, OR = Odds ratio, CI = Confidence interval

## Discussion

Gross fetal movement above the 90th percentile on the reference curve, as counted by a fetal movement acceleration measurement recorder over 4 h, was not related to abnormal perinatal outcomes. On the contrary, it had a protective effect against preterm birth, fetal non-cephalic presentation at delivery, and Cesarean delivery. It seemed that women who showed an increased movement parameter at least once usually had relatively increased fetal movement. In addition, the background characteristics showed that the study group included more multipara and women with lower BMI.

An increase in fetal movement was related to fewer preterm births. Our hospital is a maternal perinatal center which treats high-risk pregnancies with a higher preterm rate. The preterm birth rate in the control group was 15.5 %, which was more than the Japanese average of 5–6 %; however, only one out of 74 women (1.4 %) in the study group delivered preterm. In the study group, the average number of available records per woman and delivery weeks were a bit higher than those in the control group. We assumed that this was partly because of the lower preterm birth rate in the study group.

Though the precise mechanism of preterm labor in humans is still unknown, one hypothesis is that a deteriorated fetal status, leading to stress, might be an essential background condition for preterm birth [13]. The onset of labor is initiated by signals originating from the fetus in some animals, and one of the most important signals is an increase in cortisol, which is a response to stress [14]. We imagine that an increase in fetal movement indicates less stress, which leads to full-term birth even in human beings. We think this an interesting viewpoint, but further studies are needed before concluding whether this is true.

The results also showed that non-cephalic presentation at birth was lower in the study group. We have two theories about this result. One theory is that fetuses with non-cephalic presentation do not move as frequently. Gross fetal movement includes leg movement, and we think there may be insufficient space preventing leg movement when a fetus is in breech presentation. The other theory is that gross fetal movements affect fetal presentations and that additional movement encourages stability. The more the gross movement, the more the possibilities to become stable head presentation. In any case, this relationship between fetal presentation and fetal movement was a new finding.

In addition, increased fetal movement had a protective effect against Cesarean delivery. Contrary to decreased movement as a negative indicator, increased movement indicated fetal well-being and reduced risk of non-reassuring fetal status leading to emergent Cesarean delivery, as was non-cephalic presentation leading to planned Cesarean delivery.

As a background factor, increased fetal movement occurred more frequently in multipara. It is well known that the birth weight of younger children is greater than that of a first child. There might be a difference of intrauterine environments between a first child and subsequent children. In addition, increased fetal movement occurred more frequently in women with lower BMI. This result is difficult for us to explain; however, we can imagine that the mother’s thinner abdominal wall, which has a lighter burden on the adjacent uterine wall, might mean more intrauterine space for a fetus to move.

Increased fetal movement was not related to abnormal perinatal outcomes in this study. The results were consistent with the reports [7], [8], [9] that showed maternal perception of increased fetal movement was not associated with adverse neonatal outcomes. However, at first glance, our results seemed to contradict reports [3], [4], [5], [6] indicating that maternal perception of increased fetal movement was associated with stillbirth. These reports, however, focused on unusually acute and excessive movement, which is much different from our fetal movement parameter which was an average value over more than four hours. Stacey et al [15]. reported that a single episode of increased fetal movement indicated a risk of stillbirth but that repeated episodes of increased movement protected against stillbirth. Heazell et al [16]. reported the same findings. These repeated episodes of increased movement might be closer to the increased movement found in our study. First, there was only one case of stillbirth in this study, which prevents any such discussion. The results of this study, therefore, cannot refute concerns that maternal perception of unusual increased fetal movement is associated with stillbirth. Further studies are needed from the viewpoint that there might be a difference between unusually acute and excessive movement and increased movement as an average value.

One of the strengths of this study was that the newly developed FMAM recorder enabled hours of objective counting of gross fetal movement. This was a first report studying the overall relationship between perinatal outcomes and increased fetal movement as counted by an objective method for many hours.

One of the limitations of this study was the relatively small number of participating women. The FMAM recorder is now available only for research and not for clinical use, and there are currently a limited number of FMAM recorders. Only six women per year can use one recorder because it takes two months to count fetal movement from 28 weeks to term. This study was translocational and still preliminary. Another limitation was that the FMAM recorder is unreliable before 28 weeks; therefore, this study lacked data about earlier periods during pregnancy.

In conclusion, an increase in gross fetal movement over many hours has a protective effect against preterm birth, non-cephalic presentation at delivery, and Cesarian delivery. Increased fetal movement over many hours is a good sign in prenatal medicine.

## CRediT authorship contribution statement

**Takashi Kosaka:** Methodology, Investigation. **Kazunori Nagasaka:** Funding acquisition. **Eiji Ryo:** Writing – original draft, Supervision, Methodology, Funding acquisition, Conceptualization. **Kamata HIdeo:** Writing – review & editing, Visualization, Validation, Project administration, Methodology, Investigation, Formal analysis, Data curation. **Keita Yatsuki:** Methodology, Investigation, Data curation.

## Ethical/institutional review board approval

This study was approved by the ethics committee of Teikyo University (13−100).

## Consent to publish

All steps were performed in accordance with relevant guidelines, and all women gave written informed consent before participating in the study.

## Funding

This work was supported by the 10.13039/501100001691Japan Society for the Promotion of Science (Grant Number JP 16K10109, Principal Investigator Eiji Ryo).

## Declaration of Competing interest

The authors declare that they have no known competing financial interests or personal relationships that could have appeared to influence the work reported in this paper.
